# 

**Published:** 1880-06

**Authors:** 


### CONTENTS:

ART. I.—Memorial of Herr Theodore Mehlhardt. Translated by C. H. E. Obermuller, D. D. S. .... 49
ART. II.—What Advantages the American Colleges of Dentistry afford the Students and the Afflicted Public. By Dr. F. W. Rudolph, Hamburg, Germany .... 53
ART. III.—Alveolar Abscess. By M. F Finley, D. D. S. .... 58
ART. IV.—Proper Management of the Proximate Cutting and Grinding Surfaces of the Teeth. By Dr. George B. McDonald .... 63
ART. V.—Dental Caries. By Dr. Louis R. Ebert .... 68
ART. VI.—Necrosis. By Dr. H. H. Townsend .... 72
ART. VII.—Bromide of Ethyl .... 76
ART. VIII.—Aconitia in Neuralgia .... 79
ART. IX.—The Natural Dentine for Capping Exposed Pulps. By Dr. Thomas C. Stellwagen .... 82
ART. X —Effects of Smoking on the Teeth. By Mr. Hepburn .... 83
ART. XI.—The Treatment of Neuralgia .... 85

### EDITORIAL, ETC.

The Suppression of Fraudulent Diplomas .... 87
Is It Found?—"Naboli" .... 89
Is Expansion a Cause of Leakage in Fillings? .... 91
At Last .... 92
Important Notice .... 93

### MONTHLY SUMMARY.

Cure of a Traumatic Salivary Fistula .... 94
The Use and Abuse of Tobacco .... 95
The Value of Revaccination .... 96

